# 2-[2-(3-Methyl­but­oxy)-5-nitro­benz­amido]­acetic acid dimethyl sulfoxide monosolvate

**DOI:** 10.1107/S1600536812027316

**Published:** 2012-06-23

**Authors:** Yun-Xia Yang, Seik Weng Ng

**Affiliations:** a Key Laboratory of Polymer Materials of Gansu Province Ministry of Education, College of Chemistry and Chemical Engineering, Northwest Normal University, Lanzhou 730070, Gansu, People’s Republic of China; b Department of Chemistry, University of Malaya, 50603 Kuala Lumpur, Malaysia; c Chemistry Department, King Abdulaziz University, PO Box 80203 Jeddah, Saudi Arabia

## Abstract

In the title compound, C_14_H_18_N_2_O_6_·C_2_H_6_OS, the –C(O)NHCH_2_CO_2_H and –O(CH_2_)_2_CH(CH_3_)_2_ substitutents of the aromatic ring are positioned such that the –NH– group is hydrogen-bond donor to the ether O atom of the other substituent. The dimethyl sulfoxide solvent mol­ecule is linked to the carb­oxy­lic acid group by an O—H⋯O hydrogen bond.

## Related literature

For background to this study, see: Shaginian *et al.* (2009 ▶).
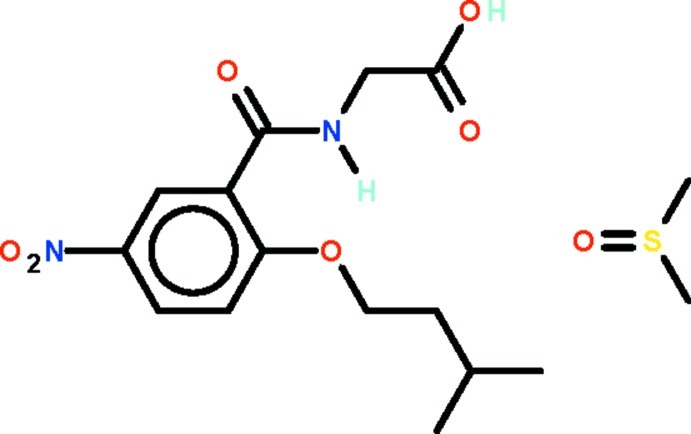


## Experimental

### 

#### Crystal data

C_14_H_18_N_2_O_6_·C_2_H_6_OS
*M* *_r_* = 388.43
Triclinic, 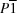

*a* = 7.4603 (12) Å
*b* = 11.2881 (18) Å
*c* = 11.5861 (19) Å
α = 99.229 (3)°
β = 99.802 (3)°
γ = 93.487 (3)°
*V* = 945.1 (3) Å^3^
*Z* = 2
Mo *K*α radiation
μ = 0.21 mm^−1^
*T* = 293 K
0.27 × 0.24 × 0.18 mm

#### Data collection

Bruker SMART APEX diffractometer
Absorption correction: multi-scan (*SADABS*; Sheldrick, 1996 ▶) *T*_min_ = 0.945, *T*_max_ = 0.963
5211 measured reflections
3644 independent reflections
2558 reflections with *I* > 2σ(*I*)
*R*_int_ = 0.035

#### Refinement

*R*[*F* ^2^ > 2σ(*F* ^2^)] = 0.057
*wR*(*F* ^2^) = 0.144
*S* = 1.01
3644 reflections
243 parameters
2 restraints
H atoms treated by a mixture of independent and constrained refinement
Δρ_max_ = 0.32 e Å^−3^
Δρ_min_ = −0.29 e Å^−3^

### 

Data collection: *APEX2* (Bruker, 2007 ▶); cell refinement: *SAINT* (Bruker, 2007 ▶); data reduction: *SAINT*; program(s) used to solve structure: *SHELXS97* (Sheldrick, 2008 ▶); program(s) used to refine structure: *SHELXL97* (Sheldrick, 2008 ▶); molecular graphics: *X-SEED* (Barbour, 2001 ▶); software used to prepare material for publication: *publCIF* (Westrip, 2010 ▶).

## Supplementary Material

Crystal structure: contains datablock(s) global, I. DOI: 10.1107/S1600536812027316/xu5567sup1.cif

Structure factors: contains datablock(s) I. DOI: 10.1107/S1600536812027316/xu5567Isup2.hkl

Supplementary material file. DOI: 10.1107/S1600536812027316/xu5567Isup3.cml

Additional supplementary materials:  crystallographic information; 3D view; checkCIF report

## Figures and Tables

**Table 1:** Hydrogen-bond geometry (Å, °)

*D*—H⋯*A*	*D*—H	H⋯*A*	*D*⋯*A*	*D*—H⋯*A*
O1—H1⋯O7	0.84 (1)	1.75 (1)	2.579 (3)	170 (3)
N1—H2⋯O4	0.87 (1)	1.97 (2)	2.656 (3)	135 (2)

**Fig. 1.**
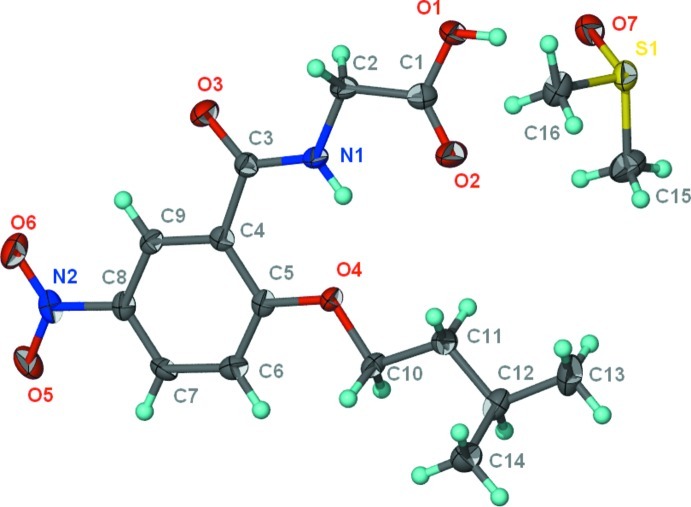
Thermal ellipsoid plot (Barbour, 2001) of C14H18N2O6.DMSO at the 70% probability level; hydrogen atoms are drawn as spheres of arbitrary radius.

## supplementary crystallographic information

### Comment

The title compound is one that is similar to those identified for a study of protein-protein interactions (Shaginian *et al.*, 2009). It crystallizes from DMSO as a monosolvate (Scheme I, Fig. 1). The –C(O)NHCH_2_CO_2_H and –O(CH_2_)_2_CH(CH_3_)_2_ substitutents of the aromatic ring of C_14_H_18_N_2_O_6_^.^DMSO are positioned such that the –NH– group is hydrogen-bond donor to the ether O of the other substituent. The solvent molecule is linked to the carboxylic acid by an O–H···O hydrogen bond (Table 1).

### Experimental

2-(Isopentyloxy)-4-nitrobenzoic acid (0.51 g, 2 mmol) was dissolved in thionyl chloride (2 ml). The synthesis is based on that reported for 2-methoxy-4-nitrobenzoic acid. The mixture was heated for half an hour. The excess reactant was evaporated and the residue dissolved in dichloromethane (10 ml) for the subsequent coupling reaction.

To a solution of methyl 2-aminoacetate hydrochloride (0.26 g, 2.1 mmol) in dichloromethane (30 ml) and triethylamine (0.5 ml) was added the above acid chloride at 273 K. The mixture was stirred at room temperature for half an hour. The solvent was removed and the residue was dissolved in methanol (50 ml) containing lithium hydroxide hydrate (0.65 g, 10 mmol) dissolved in water (2 ml). The mixture was heated for 5 h. The residue after removal of most of the solvent was acidified with 10% hydrochloric acid to a pH of 3. Filtration afforded the product as a white solid (0.41 g, 70%). Crystals were grown in DMSO.

### Refinement

Carbon-bound H-atoms were placed in calculated positions (C–H 0.93–0.97 Å) and were included in the refinement in the riding model approximation, with *U*(H) set to 1.20 to 1.5*U*(C).

The acid and amino H-atoms were located in a difference Fourier map, and were refined with distance restrainst of O–H 0.84±0.01 and N–H 0.84±0.01 Å; their temperature factors were freely refined.

### Crystal data

**Table:**

C_14_H_18_N_2_O_6_·C_2_H_6_OS	*Z* = 2
*M**_r_* = 388.43	*F*(000) = 412
Triclinic, *P*1	*D*_x_ = 1.365 Mg m^−^^3^
Hall symbol: -P 1	Mo *K*α radiation, λ = 0.71073 Å
*a* = 7.4603 (12) Å	Cell parameters from 3644 reflections
*b* = 11.2881 (18) Å	θ = 1.8–26.0°
*c* = 11.5861 (19) Å	µ = 0.21 mm^−^^1^
α = 99.229 (3)°	*T* = 293 K
β = 99.802 (3)°	Prism, colorless
γ = 93.487 (3)°	0.27 × 0.24 × 0.18 mm
*V* = 945.1 (3) Å^3^	

### Data collection

**Table:**

Bruker SMART APEX diffractometer	3644 independent reflections
Radiation source: fine-focus sealed tube	2558 reflections with *I* > 2σ(*I*)
Graphite monochromator	*R*_int_ = 0.035
ω scans	θ_max_ = 26.0°, θ_min_ = 1.8°
Absorption correction: multi-scan (*SADABS*; Sheldrick, 1996)	*h* = −8→9
*T*_min_ = 0.945, *T*_max_ = 0.963	*k* = −12→13
5211 measured reflections	*l* = −13→14

### Refinement

**Table:**

Refinement on *F*^2^	Primary atom site location: structure-invariant direct methods
Least-squares matrix: full	Secondary atom site location: difference Fourier map
*R*[*F*^2^ > 2σ(*F*^2^)] = 0.057	Hydrogen site location: inferred from neighbouring sites
*wR*(*F*^2^) = 0.144	H atoms treated by a mixture of independent and constrained refinement
*S* = 1.01	*w* = 1/[σ^2^(*F*_o_^2^) + (0.0647*P*)^2^] where *P* = (*F*_o_^2^ + 2*F*_c_^2^)/3
3644 reflections	(Δ/σ)_max_ = 0.001
243 parameters	Δρ_max_ = 0.32 e Å^−^^3^
2 restraints	Δρ_min_ = −0.29 e Å^−^^3^

### Fractional atomic coordinates and isotropic or equivalent isotropic displacement parameters (Å^2^)

**Table:**

	*x*	*y*	*z*	*U*_iso_*/*U*_eq_	
S1	0.28957 (10)	−0.17297 (6)	0.17669 (6)	0.0234 (2)	
O1	0.2603 (3)	−0.00955 (17)	0.47071 (18)	0.0255 (5)	
O2	0.2747 (3)	0.14280 (17)	0.36944 (18)	0.0370 (6)	
O3	0.1140 (3)	0.36605 (17)	0.72134 (17)	0.0262 (5)	
O4	0.2827 (2)	0.47573 (16)	0.42855 (15)	0.0181 (4)	
O5	0.1885 (3)	0.92011 (17)	0.80371 (18)	0.0297 (5)	
O6	0.0932 (3)	0.77455 (18)	0.88517 (17)	0.0295 (5)	
O7	0.3687 (3)	−0.15722 (17)	0.30829 (17)	0.0271 (5)	
N1	0.2100 (3)	0.3040 (2)	0.5503 (2)	0.0187 (5)	
N2	0.1524 (3)	0.8122 (2)	0.8040 (2)	0.0222 (6)	
C1	0.2487 (4)	0.1034 (2)	0.4564 (2)	0.0208 (6)	
C2	0.1965 (4)	0.1786 (2)	0.5629 (2)	0.0194 (6)	
H2A	0.0725	0.1533	0.5692	0.023*	
H2B	0.2772	0.1679	0.6347	0.023*	
C3	0.1688 (4)	0.3899 (2)	0.6327 (2)	0.0167 (6)	
C4	0.1942 (3)	0.5189 (2)	0.6155 (2)	0.0153 (6)	
C5	0.2520 (3)	0.5602 (2)	0.5177 (2)	0.0155 (6)	
C6	0.2746 (4)	0.6832 (2)	0.5155 (2)	0.0191 (6)	
H6	0.3113	0.7095	0.4504	0.023*	
C7	0.2429 (4)	0.7664 (2)	0.6094 (2)	0.0192 (6)	
H7	0.2603	0.8486	0.6089	0.023*	
C8	0.1850 (4)	0.7253 (2)	0.7040 (2)	0.0181 (6)	
C9	0.1608 (3)	0.6042 (2)	0.7080 (2)	0.0153 (6)	
H9	0.1217	0.5794	0.7731	0.018*	
C10	0.3186 (4)	0.5110 (2)	0.3185 (2)	0.0181 (6)	
H10A	0.4303	0.5641	0.3334	0.022*	
H10B	0.2187	0.5524	0.2832	0.022*	
C11	0.3368 (4)	0.3956 (2)	0.2372 (2)	0.0182 (6)	
H11A	0.2246	0.3435	0.2253	0.022*	
H11B	0.4346	0.3547	0.2757	0.022*	
C12	0.3764 (4)	0.4145 (3)	0.1161 (2)	0.0232 (7)	
H12	0.4864	0.4705	0.1289	0.028*	
C13	0.4120 (4)	0.2954 (3)	0.0449 (3)	0.0322 (8)	
H13A	0.4378	0.3084	−0.0307	0.048*	
H13B	0.3061	0.2391	0.0328	0.048*	
H13C	0.5146	0.2637	0.0878	0.048*	
C14	0.2198 (4)	0.4682 (3)	0.0460 (3)	0.0283 (7)	
H14A	0.1978	0.5432	0.0912	0.043*	
H14B	0.1117	0.4133	0.0307	0.043*	
H14C	0.2511	0.4820	−0.0280	0.043*	
C15	0.3742 (5)	−0.0420 (3)	0.1283 (3)	0.0330 (8)	
H15A	0.5024	−0.0447	0.1276	0.049*	
H15B	0.3552	0.0286	0.1816	0.049*	
H15C	0.3105	−0.0393	0.0496	0.049*	
C16	0.0586 (4)	−0.1409 (3)	0.1671 (3)	0.0285 (7)	
H16A	−0.0098	−0.2046	0.1910	0.043*	
H16B	0.0083	−0.1345	0.0866	0.043*	
H16C	0.0520	−0.0662	0.2185	0.043*	
H1	0.295 (4)	−0.051 (2)	0.4133 (19)	0.035 (10)*	
H2	0.242 (4)	0.323 (2)	0.4863 (16)	0.027 (9)*	

### Atomic displacement parameters (Å^2^)

**Table:**

	*U*^11^	*U*^22^	*U*^33^	*U*^12^	*U*^13^	*U*^23^
S1	0.0284 (4)	0.0195 (4)	0.0233 (4)	0.0026 (3)	0.0109 (3)	0.0000 (3)
O1	0.0410 (14)	0.0135 (11)	0.0255 (12)	0.0049 (10)	0.0135 (10)	0.0049 (9)
O2	0.0716 (18)	0.0176 (12)	0.0282 (12)	0.0052 (11)	0.0252 (12)	0.0055 (9)
O3	0.0385 (13)	0.0217 (11)	0.0219 (11)	0.0012 (10)	0.0145 (10)	0.0051 (9)
O4	0.0240 (11)	0.0174 (10)	0.0149 (10)	0.0030 (8)	0.0083 (8)	0.0034 (8)
O5	0.0378 (13)	0.0145 (11)	0.0364 (13)	0.0038 (10)	0.0104 (10)	−0.0012 (9)
O6	0.0344 (13)	0.0317 (13)	0.0230 (12)	−0.0004 (10)	0.0142 (10)	−0.0013 (9)
O7	0.0350 (13)	0.0226 (12)	0.0238 (11)	0.0060 (10)	0.0064 (9)	0.0019 (9)
N1	0.0255 (14)	0.0140 (12)	0.0190 (13)	0.0003 (10)	0.0092 (11)	0.0049 (10)
N2	0.0197 (13)	0.0208 (14)	0.0245 (14)	0.0040 (11)	0.0047 (11)	−0.0023 (11)
C1	0.0210 (16)	0.0204 (16)	0.0208 (16)	−0.0004 (13)	0.0026 (12)	0.0053 (13)
C2	0.0232 (16)	0.0152 (15)	0.0209 (15)	−0.0016 (12)	0.0066 (12)	0.0053 (12)
C3	0.0160 (14)	0.0173 (15)	0.0169 (14)	0.0025 (12)	0.0028 (11)	0.0032 (12)
C4	0.0121 (14)	0.0172 (15)	0.0162 (14)	0.0019 (11)	0.0004 (11)	0.0033 (11)
C5	0.0106 (14)	0.0202 (15)	0.0145 (14)	0.0019 (11)	0.0003 (11)	0.0016 (11)
C6	0.0186 (15)	0.0209 (16)	0.0182 (15)	0.0006 (12)	0.0022 (12)	0.0066 (12)
C7	0.0216 (16)	0.0126 (14)	0.0243 (16)	0.0041 (12)	0.0038 (12)	0.0055 (12)
C8	0.0163 (15)	0.0199 (15)	0.0169 (15)	0.0025 (12)	0.0026 (11)	−0.0004 (12)
C9	0.0100 (13)	0.0210 (15)	0.0154 (14)	0.0018 (11)	0.0027 (11)	0.0046 (11)
C10	0.0210 (15)	0.0214 (15)	0.0130 (14)	0.0017 (12)	0.0055 (11)	0.0040 (11)
C11	0.0153 (15)	0.0195 (15)	0.0206 (15)	0.0020 (12)	0.0040 (11)	0.0053 (12)
C12	0.0207 (16)	0.0300 (18)	0.0195 (16)	−0.0003 (13)	0.0075 (12)	0.0029 (13)
C13	0.0303 (18)	0.045 (2)	0.0211 (16)	0.0127 (16)	0.0077 (14)	−0.0016 (14)
C14	0.0378 (19)	0.0287 (18)	0.0197 (16)	0.0055 (15)	0.0064 (14)	0.0058 (13)
C15	0.039 (2)	0.0322 (19)	0.0277 (18)	−0.0078 (16)	0.0114 (15)	0.0055 (14)
C16	0.0275 (18)	0.0353 (19)	0.0238 (16)	0.0034 (14)	0.0088 (13)	0.0041 (14)

### Geometric parameters (Å, º)

**Table:**

S1—O7	1.516 (2)	C7—H7	0.9300
S1—C16	1.771 (3)	C8—C9	1.378 (4)
S1—C15	1.781 (3)	C9—H9	0.9300
O1—C1	1.317 (3)	C10—C11	1.507 (4)
O1—H1	0.838 (10)	C10—H10A	0.9700
O2—C1	1.205 (3)	C10—H10B	0.9700
O3—C3	1.230 (3)	C11—C12	1.526 (4)
O4—C5	1.348 (3)	C11—H11A	0.9700
O4—C10	1.457 (3)	C11—H11B	0.9700
O5—N2	1.232 (3)	C12—C14	1.521 (4)
O6—N2	1.228 (3)	C12—C13	1.524 (4)
N1—C3	1.336 (3)	C12—H12	0.9800
N1—C2	1.445 (3)	C13—H13A	0.9600
N1—H2	0.873 (10)	C13—H13B	0.9600
N2—C8	1.457 (3)	C13—H13C	0.9600
C1—C2	1.504 (4)	C14—H14A	0.9600
C2—H2A	0.9700	C14—H14B	0.9600
C2—H2B	0.9700	C14—H14C	0.9600
C3—C4	1.505 (4)	C15—H15A	0.9600
C4—C9	1.389 (3)	C15—H15B	0.9600
C4—C5	1.414 (3)	C15—H15C	0.9600
C5—C6	1.394 (4)	C16—H16A	0.9600
C6—C7	1.381 (4)	C16—H16B	0.9600
C6—H6	0.9300	C16—H16C	0.9600
C7—C8	1.379 (4)		
			
O7—S1—C16	106.07 (13)	O4—C10—H10A	110.6
O7—S1—C15	105.75 (13)	C11—C10—H10A	110.6
C16—S1—C15	98.07 (15)	O4—C10—H10B	110.6
C1—O1—H1	112 (2)	C11—C10—H10B	110.6
C5—O4—C10	119.82 (19)	H10A—C10—H10B	108.7
C3—N1—C2	121.4 (2)	C10—C11—C12	113.6 (2)
C3—N1—H2	119.6 (19)	C10—C11—H11A	108.8
C2—N1—H2	119.0 (19)	C12—C11—H11A	108.8
O6—N2—O5	123.2 (2)	C10—C11—H11B	108.8
O6—N2—C8	118.6 (2)	C12—C11—H11B	108.8
O5—N2—C8	118.2 (2)	H11A—C11—H11B	107.7
O2—C1—O1	125.2 (3)	C14—C12—C13	109.7 (2)
O2—C1—C2	123.4 (2)	C14—C12—C11	111.6 (2)
O1—C1—C2	111.4 (2)	C13—C12—C11	110.1 (2)
N1—C2—C1	109.4 (2)	C14—C12—H12	108.4
N1—C2—H2A	109.8	C13—C12—H12	108.4
C1—C2—H2A	109.8	C11—C12—H12	108.4
N1—C2—H2B	109.8	C12—C13—H13A	109.5
C1—C2—H2B	109.8	C12—C13—H13B	109.5
H2A—C2—H2B	108.2	H13A—C13—H13B	109.5
O3—C3—N1	121.8 (2)	C12—C13—H13C	109.5
O3—C3—C4	120.2 (2)	H13A—C13—H13C	109.5
N1—C3—C4	118.1 (2)	H13B—C13—H13C	109.5
C9—C4—C5	118.1 (2)	C12—C14—H14A	109.5
C9—C4—C3	115.2 (2)	C12—C14—H14B	109.5
C5—C4—C3	126.7 (2)	H14A—C14—H14B	109.5
O4—C5—C6	122.6 (2)	C12—C14—H14C	109.5
O4—C5—C4	117.1 (2)	H14A—C14—H14C	109.5
C6—C5—C4	120.3 (2)	H14B—C14—H14C	109.5
C7—C6—C5	120.5 (2)	S1—C15—H15A	109.5
C7—C6—H6	119.7	S1—C15—H15B	109.5
C5—C6—H6	119.7	H15A—C15—H15B	109.5
C8—C7—C6	118.8 (2)	S1—C15—H15C	109.5
C8—C7—H7	120.6	H15A—C15—H15C	109.5
C6—C7—H7	120.6	H15B—C15—H15C	109.5
C9—C8—C7	121.9 (2)	S1—C16—H16A	109.5
C9—C8—N2	118.8 (2)	S1—C16—H16B	109.5
C7—C8—N2	119.3 (2)	H16A—C16—H16B	109.5
C8—C9—C4	120.4 (2)	S1—C16—H16C	109.5
C8—C9—H9	119.8	H16A—C16—H16C	109.5
C4—C9—H9	119.8	H16B—C16—H16C	109.5
O4—C10—C11	105.9 (2)		
			
C3—N1—C2—C1	179.2 (2)	C4—C5—C6—C7	0.8 (4)
O2—C1—C2—N1	−8.4 (4)	C5—C6—C7—C8	−1.3 (4)
O1—C1—C2—N1	172.2 (2)	C6—C7—C8—C9	1.0 (4)
C2—N1—C3—O3	−1.5 (4)	C6—C7—C8—N2	179.6 (2)
C2—N1—C3—C4	177.4 (2)	O6—N2—C8—C9	−4.5 (4)
O3—C3—C4—C9	3.1 (4)	O5—N2—C8—C9	174.3 (2)
N1—C3—C4—C9	−175.9 (2)	O6—N2—C8—C7	176.8 (3)
O3—C3—C4—C5	−178.5 (3)	O5—N2—C8—C7	−4.3 (4)
N1—C3—C4—C5	2.5 (4)	C7—C8—C9—C4	−0.1 (4)
C10—O4—C5—C6	−8.0 (4)	N2—C8—C9—C4	−178.7 (2)
C10—O4—C5—C4	171.8 (2)	C5—C4—C9—C8	−0.5 (4)
C9—C4—C5—O4	−179.6 (2)	C3—C4—C9—C8	178.0 (2)
C3—C4—C5—O4	2.1 (4)	C5—O4—C10—C11	−177.2 (2)
C9—C4—C5—C6	0.2 (4)	O4—C10—C11—C12	−179.6 (2)
C3—C4—C5—C6	−178.1 (3)	C10—C11—C12—C14	−63.7 (3)
O4—C5—C6—C7	−179.5 (2)	C10—C11—C12—C13	174.2 (2)

### Hydrogen-bond geometry (Å, º)

**Table:**

*D*—H···*A*	*D*—H	H···*A*	*D*···*A*	*D*—H···*A*
O1—H1···O7	0.84 (1)	1.75 (1)	2.579 (3)	170 (3)
N1—H2···O4	0.87 (1)	1.97 (2)	2.656 (3)	135 (2)
